# A DNA-Based Image Encryption Scheme Using Optimized Lorenz–Sprott Hyperchaotic System

**DOI:** 10.3390/e28090964

**Published:** 2026-08-27

**Authors:** Wenxia Xu, Liang Xue, Liping Zhu, Jiaofen Li, Guodong Li

**Affiliations:** 1 School of Computer Science and Information Security, Guilin University of Electronic Technology, Guilin 541004, China; xwx@guet.edu.cn; 2 School of Mathematics and Computational Science, Guilin University of Electronic Technology, Guilin 541004, China; xueliang124@126.com (L.X.); lgdzhy@126.com (G.L.); 3 School of Mathematics and Data Science, Changji University, Changji 831100, China; 4 Center for Applied Mathematics of Guangxi (GUET), Guilin 541004, China; 5 Guangxi Academy of Artificial Intelligence, Nanning 530200, China

**Keywords:** hyperchaotic system, PSO, dynamic DNA encoding, chained diffusion, image encryption

## Abstract

Chaotic systems have been widely investigated for color image encryption because of their nonlinear dynamics, initial-condition sensitivity, and pseudorandom behavior. However, locating numerically robust parameter regions in high-dimensional hyperchaotic systems remains difficult, while many DNA-based schemes employ fixed or weakly varying rules. This study proposes a color image encryption scheme combining a 6D Lorenz–Sprott system optimized by particle swarm optimization (PSO), symbol-level feedback-dependent DNA transformation, and bidirectional cross-channel chained diffusion. The second-largest Lyapunov exponent is used as the optimization objective to locate parameter sets with at least two positive exponents. The selected system has the Lyapunov spectrum (0.8118, 0.2736, −0.0013, −4.8957, −8.5499, −12.6746) and retains two positive exponents under refined numerical settings and ±1% single-parameter perturbations. One hundred independently initialized sequences satisfy all 15 categories of the NIST SP 800-22 test suite. Tests on six images and three secret keys achieve exact reconstruction in all 18 cases. Across 180 randomly located one-bit plaintext perturbations, the mean NPCR and UACI are 99.6089% and 33.4554%, respectively. For the tested Baboon case, perturbing any initial-state component by approximately 10^−14^ prevents meaningful plaintext recovery. Ablation results further demonstrate the contributions of dynamic DNA transformation and bidirectional diffusion. The proposed scheme therefore provides reproducible hyperchaotic parameter modulation and strong empirical statistical and differential performance.

## 1. Introduction

Images are widely transmitted in medical, military, and other multimedia applications, where their large data volume, spatial redundancy, and inter-channel correlation motivate image-oriented protection [1,2,3,4]. Although AES remains an established security standard, chaos-based image encryption explores permutation, diffusion, and parallel structures driven by deterministic nonlinear sequences [5,6,7,8,9,10]. The practical use of such sequences nevertheless requires independent verification of their numerical robustness and digital statistical properties.

Low-dimensional logistic and tent maps [11,12] offer low computational cost, while compound maps and compressed-sensing-assisted architectures [13,14] introduce richer nonlinear transformations. However, additional encryption operations do not by themselves overcome the limited state dimension or finite-precision degradation of the underlying generator; its randomness and numerical robustness must therefore be evaluated separately from ciphertext security metrics.

High-dimensional and hyperchaotic generators provide more state variables and may possess multiple expanding directions. Existing schemes combine them with multilayer encryption, DNA encoding, random-signal perturbation, dynamic substitution, and compressive sensing [15,16,17,18], but the larger parameter space increases numerical cost and complicates reproducibility. Coupling classical systems is one possible solution: the Lorenz system supplies a well-studied dissipative framework [19], whereas Sprott systems offer compact nonlinear structures [20]. Coupled-map and cross-feedback studies [21,22,23] show that subsystem interactions can enrich the dynamics; however, coupling alone does not guarantee a numerically robust hyperchaotic regime.

Recent studies further demonstrate the need to connect dynamical properties with application requirements. Shi et al. [24] combined a 3D exponential hyperchaotic map with an integer lifting wavelet transform to achieve lossless frequency-domain encryption, but did not address automated parameter localization in a high-dimensional continuous system or feedback-dependent RGB coupling. In a different application context, Yu et al. [25] linked the hidden extreme multistability of a five-dimensional memristive Hopfield network to LSTM-based forecasting, illustrating that complex dynamics should be evaluated according to their downstream function. These studies indicate that generator construction, parameter selection, numerical verification, and application architecture require connected but distinct validation.

The nine adjustable coefficients of the proposed coupled system create a bounded search space that is impractical for exhaustive scanning. To reduce reliance on empirical trial-and-error, PSO [26] is employed with the second-largest Lyapunov exponent as a bottleneck objective, explicitly favoring parameter sets with at least two positive exponents before long-horizon and perturbation verification.

The chaotic sequences are further integrated with DNA encoding, which maps 2-bit pixel symbols to A, C, G, and T and supports reversible substitution and XOR operations [27,28,29,30,31,32,33,34,35]. However, favorable statistical metrics do not ensure resistance to stronger attacks: Chen et al. [36] demonstrated chosen-plaintext and equivalent-key weaknesses in a chaos- and DNA-based medical image scheme. Accordingly, the standard DNA rules are not treated here as novel or as independent secret-key dimensions. The proposed design instead updates the rule and 2-bit mask using key-dependent chaotic streams and the preceding transformed symbol, and combines this mechanism with cross-channel chained diffusion to improve plaintext propagation while preserving invertibility.

This study makes three main contributions:

1. A PSO framework uses the second-largest Lyapunov exponent to identify a reproducible hyperchaotic parameter set for the 6D Lorenz–Sprott system, supported by long-horizon and numerical-robustness verification.
2. A symbol-level DNA transformation dynamically updates both the rule and mask through chaotic controls and feedback, while bidirectional cross-channel diffusion propagates changes across pixels and RGB channels.
3. Multi-sequence, multi-image, and multi-key experiments, together with key-sensitivity, ablation, and runtime analyses, characterize the scheme’s statistical performance, reproducibility, and practical limitations.

Section 2 presents the optimized 6D system and its dynamical verification. Section 3 describes the encryption and decryption procedures, Section 4 reports the security and runtime evaluations, and Section 5 concludes the paper.

## 2. 6D Lorenz–Sprott Hyperchaotic System and PSO

### 2.1. Construction of the 6D Lorenz–Sprott Hyperchaotic Model

The proposed model couples the classical Lorenz and Sprott-A systems. The Lorenz subsystem is

(1) $$\left\{\begin{array}{c} \dot{x}=\sigma \left(y-x\right),\\ \dot{y}=x(\rho -z)-y\\ \dot{z}=xy-\beta z, \end{array},\right.$$

where the standard parameters are set as σ = 10, ρ = 28, β = 8/3. The structurally compact Sprott-A subsystem is

(2) $$\left\{\begin{array}{c} \dot{u}=v,\\ \dot{v}=-u+vw,\\ \dot{w}=1-v^{2}. \end{array}\right.$$

The Lorenz subsystem supplies a dissipative framework with the quadratic terms *xz* and *xy*, whereas Sprott-A provides a compact oscillator containing the nonlinearities *vw* and *v*^2^. To prevent the resulting 6D model from remaining separable, bidirectional state injection and state-dependent parameter modulation are introduced. In the Lorenz subsystem, *w* modulates $\rho_{0}$ through $\rho_{0}$ + *αw*; *k*_1_*u* and *k*_2_*v* provide linear injection, and *k*_3_*uv* introduces nonlinear perturbation. Conversely, *λ*_1_*x*, *λ*_2_*y*, and *λ*_3_*z* feed the Lorenz states into the Sprott subsystem, while *λ*_4_*xz* provides quadratic cross-injection and −*δw* supplies adjustable damping. These asymmetric pathways form a closed feedback loop, and the coupled system is

(3) $$\left\{\begin{array}{c} \dot{x}=\sigma \left(y-x\right)+k_{1}u,\\ \dot{y}=x\left(\rho_{0}+\alpha w-z\right)-y+k_{2}v,\\ \dot{z}=xy-\beta z+k_{3}uv,\\ \begin{array}{c} \dot{u}=v+\lambda_{1}x,\\ \dot{v}=-u+vw+\lambda_{2}y+\lambda_{4}xz,\\ \dot{w}=1-v^{2}+\lambda_{3}z-\delta w, \end{array} \end{array}\right.$$

where *σ* = 10, $\rho_{0}$ = 28, and *β* = 8/3 retain the standard Lorenz values, while *α, k*_1_, *k*_2_, *k*_3_, *λ*_1_, *λ*_2_, *λ*_3_, *λ*_4_, and *δ* are adjustable parameters. The divergence is ∇·*F* = *w* − (*σ* + 1 + *β* + *δ*), indicating state-dependent contraction. For the optimized parameters in Section 2.3, the Lyapunov-exponent sum is −25.0361; thus, average phase-volume contraction coexists with expansion in two independent directions, supporting bounded and initial-state-sensitive trajectories.

The six coupled states provide nonidentical components for the permutation, diffusion, and DNA-control streams. Because hyperchaos alone does not establish cryptographic security, the optimized system is further evaluated through long-horizon Lyapunov analysis, parameter and step-size robustness, multiple-sequence NIST tests, and image-security experiments.

### 2.2. PSO-Based Adaptive Parameter Modulation Framework

The nine adjustable parameters create a bounded search space that remains too large for exhaustive scanning at a resolution of 0.1. PSO was therefore employed to locate promising hyperchaotic regions through population-based exploration.

PSO was selected because it directly represents each candidate as a bounded real-valued parameter vector, supports straightforward bound handling, retains individual and population memories through *P*_best_ and *G*_best_, and adds little overhead relative to the costly Lyapunov-exponent evaluation. In comparison, GA requires choices of encoding, selection, crossover, and mutation operators [37]; DE depends on the mutation scale, crossover rate, and population diversity [38]; and GWO may lose diversity as agents concentrate around leading solutions [39]. This comparison does not imply that PSO is universally superior, and a controlled comparison of these optimizers remains future work.

**Figure 1:**
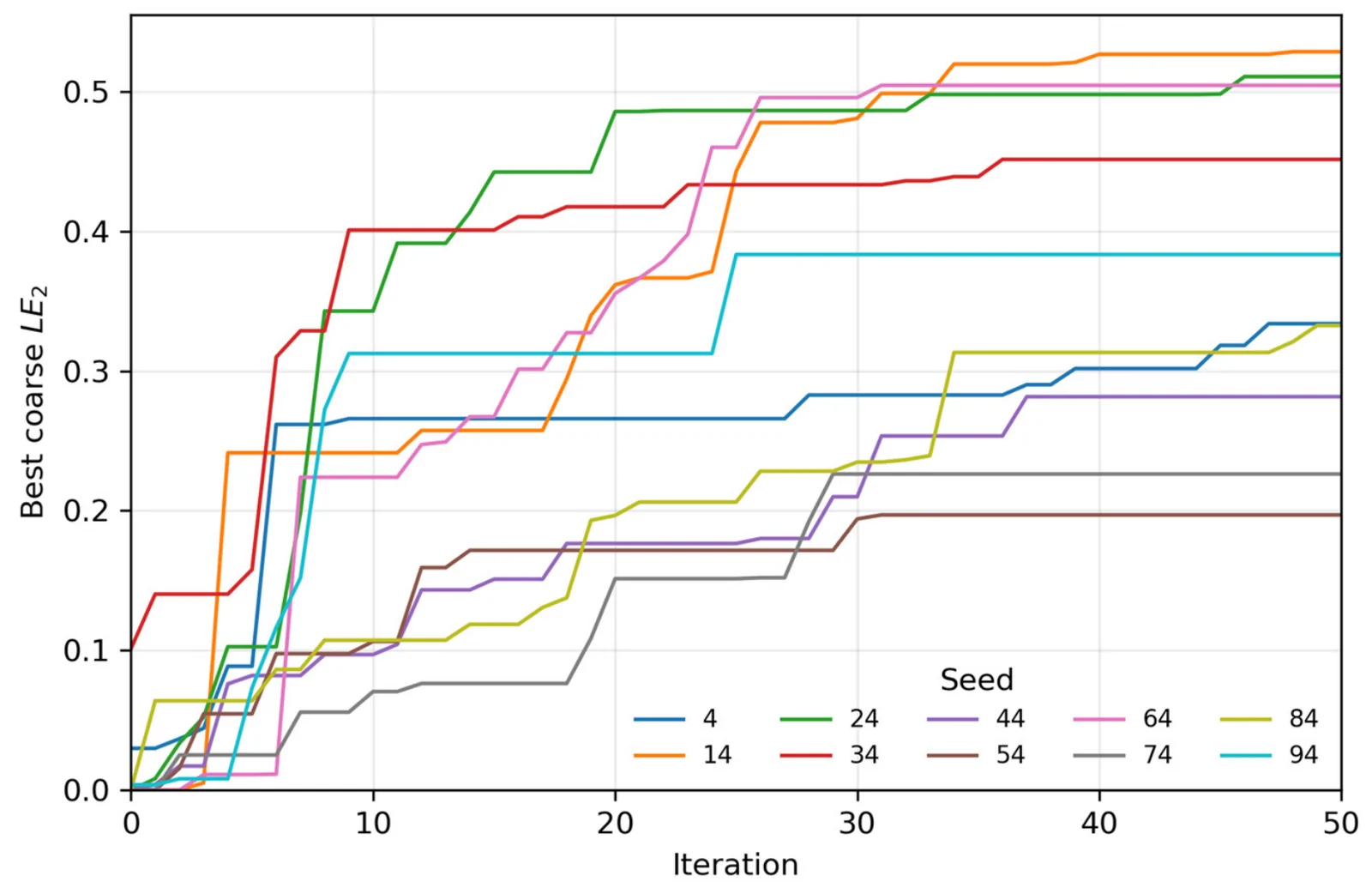
PSO convergence of the best coarse *LE*_2_ over ten independent runs.

Because the Lyapunov exponents are ordered, *LE*_2_ > 0 guarantees at least two positive exponents. Unlike maximizing *LE*_1_ + *LE*_2_, maximizing *LE*_2_ prevents a large *LE*_1_ from masking a near-zero *LE*_2_. PSO therefore minimizes

(4) $$f\left(p\right)=\left\{\begin{array}{c} -{LE}_{2}(p),\;if\;{LE}_{2}(p)>0\\ P,\;else \end{array}\right.P=100,$$

where *p* = [*α*, *k*_1_, *k*_2_, *k*_3_, *λ*_1_, *λ*_2_, *λ*_3_, *λ*_4_, *δ*]. Hyperchaotic candidates have the negative objective −*LE*_2_(*p*), whereas non-hyperchaotic or divergent candidates receive *P* = 100. Any nonnegative penalty would preserve this feasible–infeasible ordering; 100 is only an identifiable marker and does not affect the ranking of feasible particles. Stochastic convergence is evaluated using the ten independent runs shown in Figure 1.

After defining the optimization objective, each particle representing a parameter combination continuously adjusts its search velocity *V* and position *X* based on its individual historical best position *P*_best_ and the global best position *G*_best_ of the population. The velocity and position updates are

(5) $$v_{i}^{t+1}=\omega v_{i}^{t}+c_{1}r_{1}\left(P_{best,i}^{t}-X_{i}^{t}\right)+c_{2}r_{2}\left(G_{best,i}^{t}-X_{i}^{t}\right),$$

(6) $$X_{i}^{t+1}=X_{i}^{t}+V_{i}^{t+1},$$

where *w* is the inertia weight, *c*_1_ and *c*_2_ are acceleration coefficients, and *r*_1_ and *r*_2_ are independently sampled from *U*(0, 1) for each particle, parameter dimension, and iteration. In all experiments, the swarm size was 40, the maximum iteration count was 50, *w* = 0.5, and *c*_1_ = *c*_2_ = 1.5. The parameter bounds were *α* ∈ [−3, 3], *k*_1_, *k*_2_, *k*_3_ ∈ [−5, 5], *λ*_1_, *λ*_2_, *λ*_3_ ∈ [−3, 3], *λ*_4_ ∈ [−2, 2], and *δ* ∈ [0, 5]. Particle positions were initialized independently from uniform distributions over these bounds, while all initial velocities were set to zero. A fixed 50-iteration stopping criterion was adopted without early termination. Each coarse Lyapunov evaluation used the initial state (1, 1, 1, 0, 5, 0), fixed-step RK4 integration with *h* = 0.01 and *T* = 300, a discarded interval *t* < 200, and QR reorthogonalization every 10 integration steps. After each position update, candidate parameters were quantized to one decimal place according to

(7) $$X_{i}^{t+1}=\frac{round(10\cdot X_{i}^{t+1})}{10},$$

and were then clipped to the prescribed bounds. To evaluate the stochastic variability of PSO, 10 independent runs were conducted using random initial populations sampled uniformly within the parameter bounds. For each run, the five best distinct candidate parameter sets were retained for subsequent long-horizon evaluation, after which a verified shortlist underwent numerical-robustness screening.

### 2.3. Dynamical Analysis

Following the ten independent PSO runs, the five best distinct candidates from each run were retained, producing 50 candidates for long-horizon evaluation with *h* = 0.01 and *T* = 1000. Twelve candidates with comparatively large verified *LE*_2_ values were then subjected to preliminary reduced-step and ±1% parameter-perturbation screening. The final parameter set was selected according to the stability of the second positive Lyapunov exponent rather than solely according to its coarse-search fitness value. The retained vector was p* = [2.8, −0.1, −4.6, −0.9, −3.0, −3.0, 2.0, −0.8, 3.3], after which the complete *h* = 0.01, 0.005, and 0.0025 step-size tests and all 18 individual ± 1% parameter perturbations were repeated with *T* = 1000.

**Figure 2:**
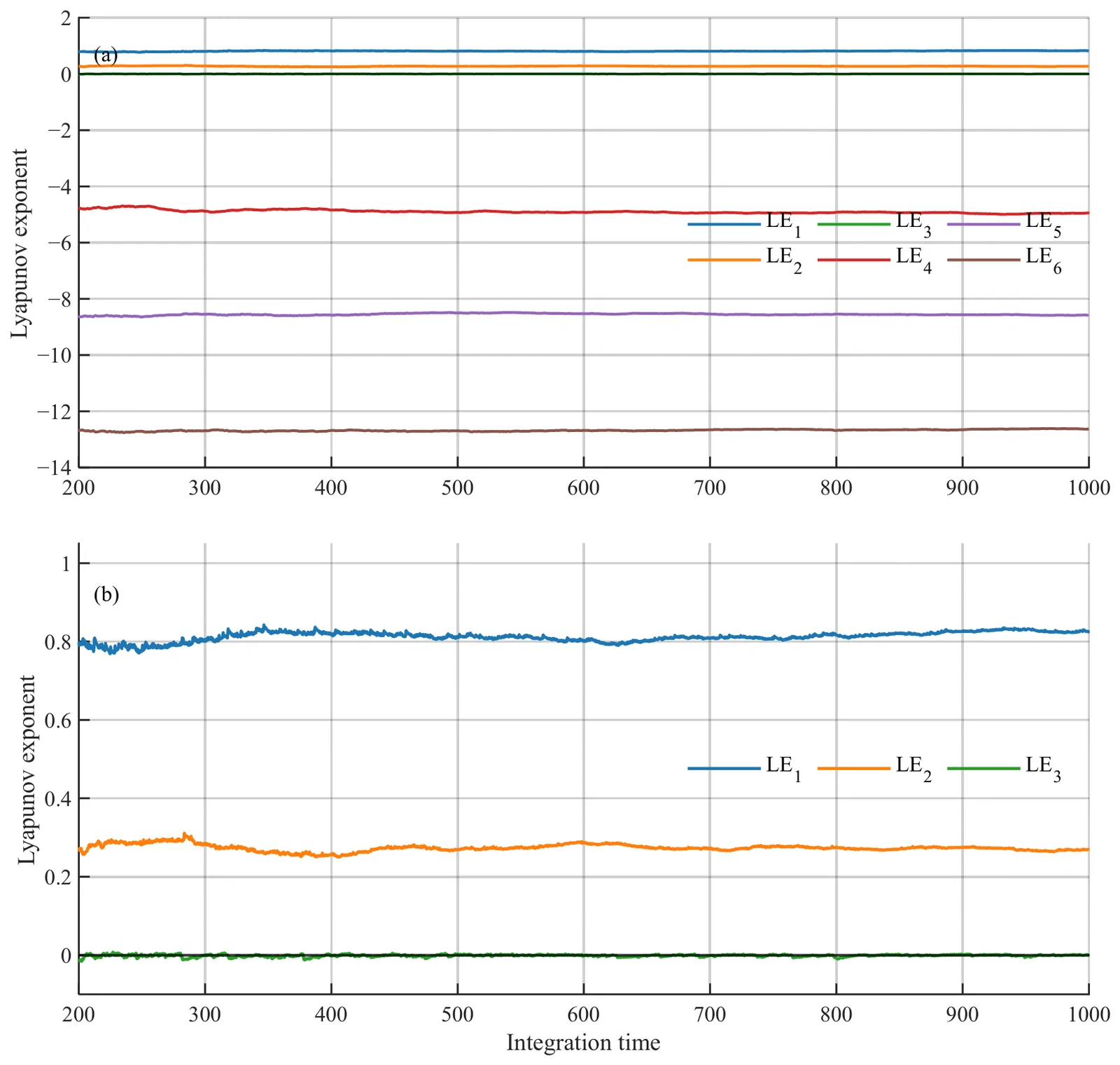
Lyapunov-exponent convergence for the optimized parameter set: (**a**) complete spectrum; (**b**) three largest exponents (*h* = 0.01, *T* = 1000).

For reproducible dynamical characterization, the initial state was fixed at (1, 1, 1, 0, 5, 0). The state and variational equations were simultaneously integrated using the fixed-step fourth-order Runge–Kutta method, with QR reorthogonalization performed every 10 integration steps. Using *h* = 0.01 and a total integration time of *T* = 1000, the running Lyapunov-exponent estimates over *t* ∈ [200, 1000] were used to obtain the complete spectrum: (*LE*_1_, *LE*_2_, *LE*_3_, *LE*_4_, *LE*_5_, *LE*_6_) = (0.8118, 0.2736, −0.0013, −4.8957, −8.5499, −12.6746). The two clearly positive exponents *LE*_1_ and *LE*_2_, together with an exponent close to zero, confirm that the optimized system operates in a hyperchaotic regime (Figure 2). The same fixed-step RK4 method and *h* = 0.01 are subsequently used for digital keystream generation in Section 3.

Numerical robustness was further examined by reducing the integration step size while keeping *T* = 1000. For *h* = 0.005, the complete spectrum was (0.7967, 0.2921, −0.0028, −4.8956, −8.5086, −12.6724), whereas for *h* = 0.0025, it was (0.8199, 0.2819, −0.0009, −4.9219, −8.5936, −12.6293). Both positive exponents were preserved under all three step sizes, demonstrating that the hyperchaotic classification is not an artifact of the numerical step size.

Parameter robustness was evaluated by independently perturbing each of the nine system parameters by +1% and −1% of its nominal magnitude, resulting in 18 perturbation cases. All 18 cases retained two positive Lyapunov exponents. Across these cases, *LE*_1_ ranged from 0.7787 to 0.8777 and *LE*_2_ ranged from 0.2353 to 0.3216. These results demonstrate that the identified hyperchaotic regime remains stable under small parameter perturbations.

**Figure 3:**
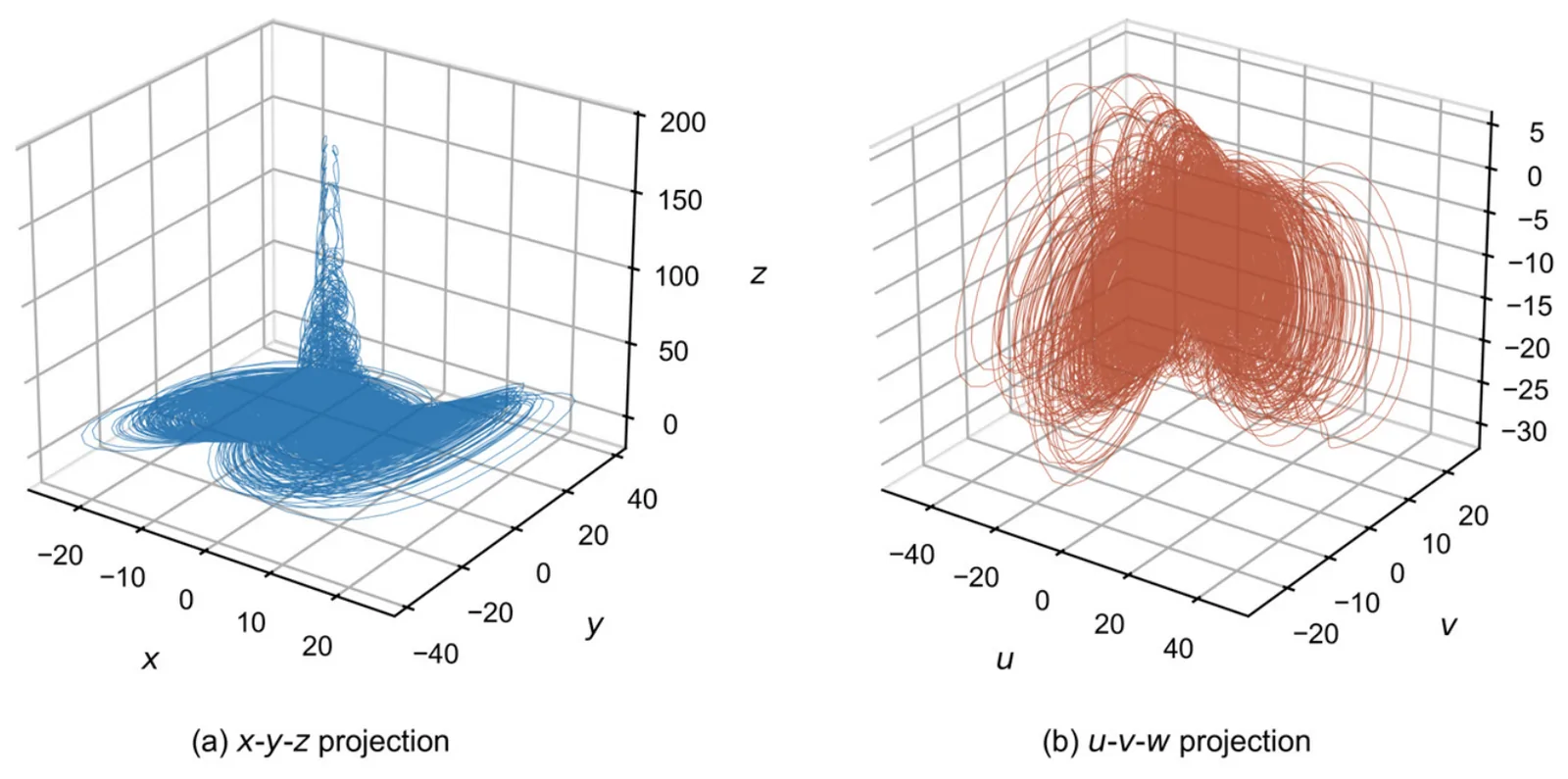
Phase portraits of the optimized 6D Lorenz–Sprott system: (**a**) *x*-*y*-*z* projection; (**b**) *u*-*v*-*w* projection.

The phase portraits obtained using the same optimized parameter set are presented in Figure 3. After excluding the initial interval *t* < 200, the trajectories in both the *x*-*y*-*z* and *u*-*v*-*w* subspaces exhibit bounded, nonperiodic, and folded attractor structures, consistent with the calculated hyperchaotic spectrum.

## 3. Image Encryption and Decryption Scheme

**Figure 4:**
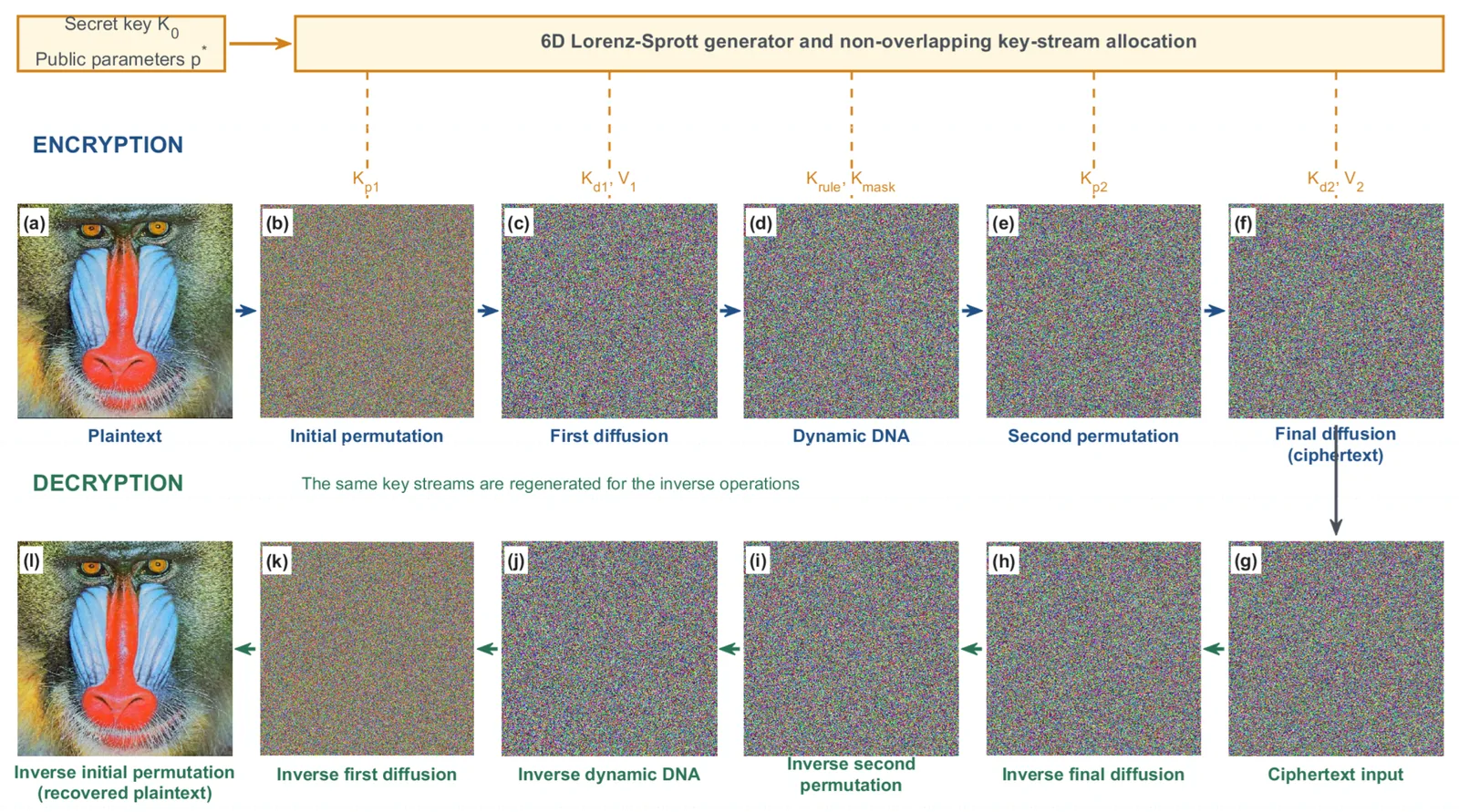
Intermediate results of the encryption and decryption processes: (**a**) plaintext; (**b**) initial permutation; (**c**) first diffusion; (**d**) dynamic DNA transformation; (**e**) second permutation; (**f**) ciphertext; (**g**) ciphertext input; (**h**) inverse final diffusion; (**i**) inverse second permutation; (**j**) inverse dynamic DNA transformation; (**k**) inverse first diffusion; and (**l**) recovered plaintext.

Based on the optimized 6D Lorenz–Sprott hyperchaotic system described above, the proposed scheme comprises initial permutation, bidirectional cross-channel diffusion, dynamic DNA transformation, secondary permutation, and final bidirectional diffusion. Decryption applies the inverse operations in reverse order, as illustrated in Figure 4.

To facilitate an unambiguous description and independent implementation, the following notation and data conventions are used throughout this section. The plaintext and ciphertext images, denoted by *P* and *C*, are RGB arrays with values in {0, …, 255} and dimensions *M* × *N* × 3, stored as unsigned 8-bit integers. Here, *M* and *N* denote the image height and width, *n* = *MN* is the number of pixels in each channel, and *L* = 3*n* is the total number of RGB samples. The channel label *ch* ∈ {*R*, *G*, *B*} is used in the permutation and diffusion equations, whereas the numeric channel index *c* ∈ {1, 2, 3}, corresponding, respectively, to *R*, *G*, and *B*, is used in the dynamic DNA transformation. The index *i* ∈ {1, …, *n*} denotes the pixel position within a vectorized channel. Each channel is vectorized in column-major order, such that *i* = *r* + (*j −* 1)*M*, where *r* ∈ {1, …, *M*} and *j* ∈ {1, …, *N*} denote the row and column indices, respectively. The chaotic state variables and normalized chaotic sequences are represented using IEEE 754 [40] double-precision numbers. All byte-level additions and subtractions are performed modulo 256; ⌊·⌋ denotes the floor operation, and ⊕ denotes bitwise XOR.

### 3.1. Encryption Algorithm

Step 1: Image Decomposition

The RGB channels are vectorized in column-major order as *P_R_*, *P_G_*, and *P_B_*, each containing *n* = *MN* unsigned 8-bit values; hence, the combined data length is *L* = 3*n*.

Step 2: Generation of 6D Chaotic Key Streams

The optimized vector p* = [2.8, −0.1, −4.6, −0.9, −3.0, −3.0, 2.0, −0.8, 3.3] is a public algorithm parameter, whereas the six-dimensional initial state *K*_0_ = (*x*_0_, *y*_0_, *z*_0_, *u*_0_, *v*_0_, *w*_0_) is the secret key. The value *K*_0_ = (1, 1, 1, 0, 5, 0) is used only as the representative experimental key.

Encryption and decryption use fixed-step RK4 with *h* = 0.01 and IEEE 754 double-precision arithmetic. The fixed step establishes a direct correspondence between iterations and state samples without adaptive-step interpolation, while the O(*h*^4^) global error of RK4 reduces numerical drift relative to the O(*h*^2^) improved Euler method. The step-size tests in Section 2.3 support *h* = 0.01 as a compromise between accuracy and efficiency. After discarding 700 iterations, the six state variables are aggregated with equal weights, and the normalized stream is

(8) $$s\left(t\right)=\frac{mod(\lfloor |\sum_{i=1}^{6}x_{i}(t)|\times 10^{9}\rfloor ,10^{9})}{10^{9}},s\left(t\right)\in [0,1).$$

The burn-in length and quantization gain are public settings rather than secret-key components. As evaluated in Section 4.7, burn-in lengths from 0 to 1000 and gains from 10^6^ to 10^9^ produced nearly unchanged entropy, bit balance, and lag-one correlation. Therefore, 700 iterations were retained as a conservative transient-removal setting rather than a mathematically optimal value. The gain 10^9^ provides the highest tested decimal resolution while satisfying the exact-integer range of double precision. Because trajectories are rejected when |*x_j_*| > 10^6^, the relevant precision bound is

(9) $$\left|\sum_{j=1}^{6}x_{j}(t)\right|\times 10^{9}\leq 6\times 10^{15}<2^{53}\approx 9.007\times 10^{15}.$$

The intermediate integer is therefore below 2^53^, the largest consecutively representable integer in IEEE 754 double precision, and is also within the signed 64-bit integer range. A direct 32-bit implementation would overflow. Hardware implementations should consequently use a 64-bit accumulator or a consistently scaled fixed-point representation. Any fixed-point conversion would require renewed dynamical and statistical validation because finite-word-length arithmetic may alter the digital trajectories.

After the transient iterations have been discarded, 6*L* + 6 consecutive samples are retained from the master chaotic sequence. The first 6*L* samples define six non-overlapping sub-key streams of equal length *L* as

(10) $$\left\{\begin{array}{c} K_{p1}=s\left(1:L\right),\\ K_{d1}=s\left(L+1:2L\right),\\ K_{rule}=s\left(2L+1:3L\right),\\ K_{mask}=s\left(3L+1:4L\right),\\ K_{p2}=s\left(4L+1:5L\right),\\ K_{d2}=s\left(5L+1:6L\right), \end{array}\right.$$

The streams *K_p_*_1_ and *K_p_*_2_ are used for the two permutation stages, *K_d_*_1_ and *K_d_*_2_ for the two bidirectional diffusion stages, and *K*_rule_ and *K*_mask_ to control the dynamic DNA rules and masks, respectively. The final six samples are reserved for the three-element unsigned 8-bit initialization vectors *V*_1_ and *V*_2_, defined in Steps 4 and 7, respectively. Because both vectors are derived deterministically from the secret-key-dependent chaotic sequence, they are not independent keys and need not be transmitted separately.

Step 3: Initial Permutation

The stream *K_p_*_1_ is divided into three channel streams *k_R_*, *k_G_*, and *k_B_*. Stable ascending sorting produces the permutation indices

(11) $$I_{ch}=sort_{index\left(k_{ch}\right)},ch\in \left\{R,G,B\right\},$$

where *I_ch_* ∈ {1, …, *n*}*^n^* records the original positions of the sorted elements. Stable sorting ensures deterministic ordering when equal values occur. The plaintext channels are permuted by

(12) $$P_{ch}^{\prime }=P_{ch}\left(I_{ch}\right),ch\in \left\{R,G,B\right\}.$$

During decryption, the original order is recovered by *P_ch_*(*I_ch_*) = $P_{ch}^{\prime }$. The resulting initially permuted image is denoted by *P*_1_.

Step 4: Initial Bidirectional Chained Diffusion

After the initial permutation, bidirectional chained diffusion is applied to *P*_1_ using *K_d_*_1_. Let *n* = *MN*. The length 3*n* stream *K_d_*_1_ is divided into three channel streams *k_R_*, *k_G_*, and *k_B_*, each of length *n*. Each floating-point stream is converted into an 8-bit integer key stream by

(13) $$K_{ch}\left(i\right)=\left\lfloor 256k_{ch}\left(i\right)\right\rfloor ,ch\in \left\{R,G,B\right\},\;i=1,\ldots ,n.$$

Because *k_ch_*(*i*) ∈ [0, 1), it follows that *K_ch_*(*i*) ∈ {0, …, 255}. Rather than being fixed, the first feedback initialization vector is derived from the first three of the final six master-stream values as

(14) $$V_{1}=\left\lfloor 256[s_{6L+1},s_{6L+2},s_{6L+3}]\right\rfloor ,$$

where *L* = 3*n* and the floor operation is applied elementwise. Thus, *V*_1_ is reproducibly generated from the secret initial state.

In the forward pass, the pixels are processed from *i* = 1 to *n*. Let (*p_R_*, *p_G_*, *p_B_*) denote the feedback values, initialized by *V*_1_. The forward recurrence is

(15) $$\left\{\begin{array}{c} F_{R(i)}=mod\left(P_{R}^{\prime }\left(i\right)+K_{R}\left(i\right)+p_{R}+p_{G}+p_{B},256\right),\\ F_{G(i)}=mod\left(P_{G}^{\prime }\left(i\right)+K_{G}\left(i\right)+p_{G}+F_{R\left(i\right)},256\right),\\ F_{B(i)}=mod\left(P_{B}^{\prime }\left(i\right)+K_{B}\left(i\right)+p_{B}+F_{G\left(i\right)},256\right). \end{array}\right.$$

After each iteration, the feedback values are updated as (*p_R_*, *p_G_*, *p_B_*) = (*F_R_*(*i*), *F_G_*(*i*), *F_B_*(*i*)). This triangular cross-channel dependency allows the current red-channel result to affect the green channel and the current green-channel result to affect the blue channel.

In the backward pass, the reversed key streams ${\bar{K}}_{\mathrm{ch}}$(*i*) = *K_ch_*(*n* − *i* + 1) are used directly; no empirical XOR perturbation constant is introduced. Starting with (*q_R_*, *q_G_*, *q_B_*) = *V*_1_, the pixels are processed from *i* = *n* to 1, and the backward recurrence is

(16) $$\left\{\begin{array}{c} D_{1,R(i)}=mod\left(F_{R\left(i\right)}+{\bar{K}}_{R\left(i\right)}+q_{R}+q_{G}+q_{B},256\right),\\ D_{1,G(i)}=mod\left(F_{G\left(i\right)}+{\bar{K}}_{G\left(i\right)}+q_{G}+D_{1,R\left(i\right)},256\right),\\ D_{1,B(i)}=mod\left(F_{B\left(i\right)}+{\bar{K}}_{B\left(i\right)}+q_{B}+D_{1,G\left(i\right)},256\right). \end{array}\right.$$

**Figure 5:**
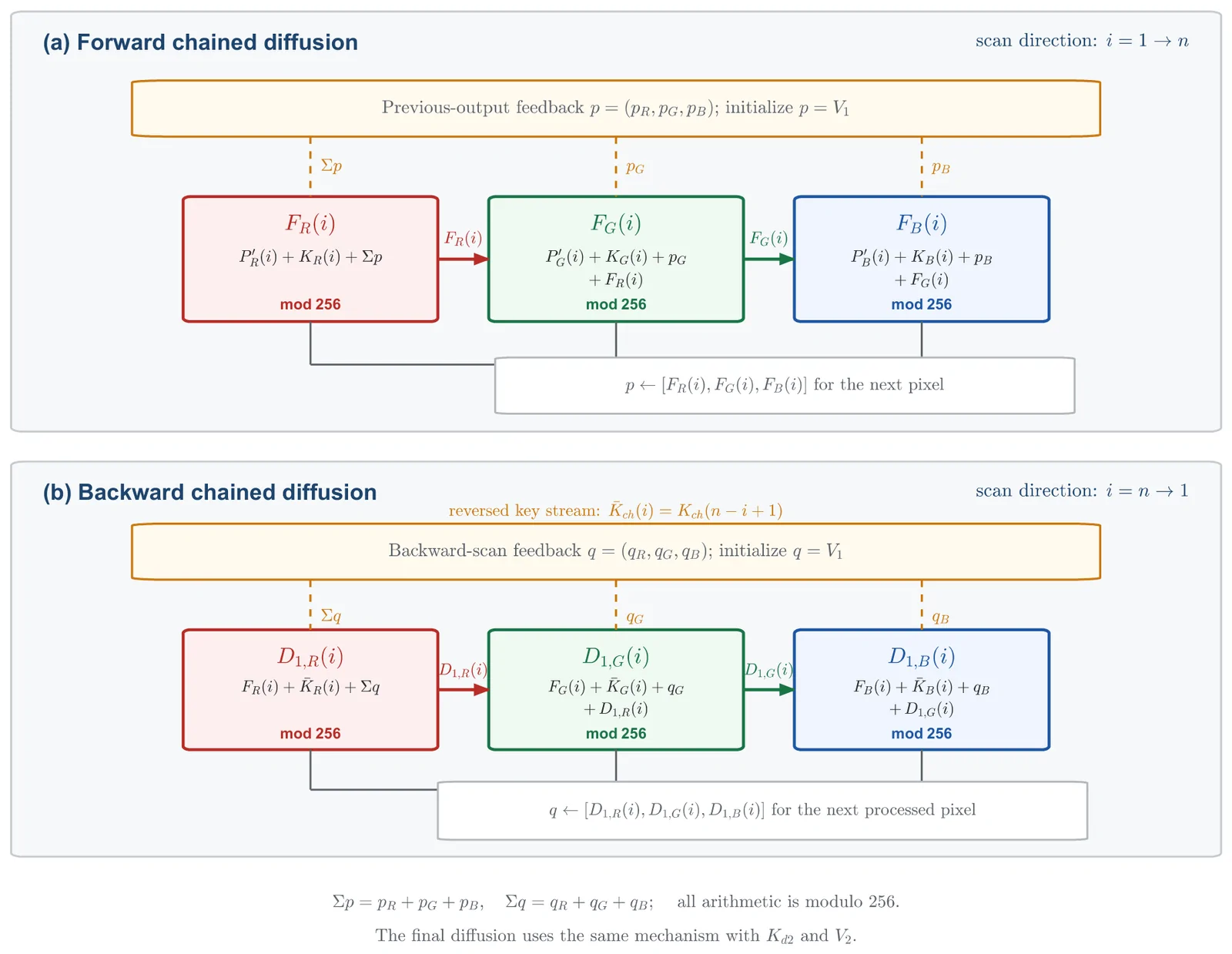
Schematic of the bidirectional chained diffusion mechanism: (**a**) forward diffusion from *i* = 1 to *n*; (**b**) backward diffusion from *i* = *n* to 1 using the reversed key streams.

After each backward iteration, (*q_R_*, *q_G_*, *q_B_*) is updated to the current output (*D*_1,*R*_(*i*), *D*_1,*G*_(*i*), *D*_1,*B*_(*i*)). The resulting image is denoted by *D*_1_, and the corresponding forward and backward feedback structures are illustrated in Figure 5.

Step 5: Dynamic DNA Encoding

Dynamic DNA transformation is subsequently applied to *D*_1_. For each channel *c* ∈ {1, 2, 3}, the corresponding length-*n* segments of *K*_rule_ and *K*_mask_ are denoted by $k_{rule}^{\left(c\right)}$ and $k_{mask}^{\left(c\right)}$, respectively. The rule-control and mask-control sequences are obtained from

(17) $$r_{i}^{\left(c\right)}=mod\left(\left\lfloor 10^{6}k_{rule}^{\left(c\right)}(i)\right\rfloor ,8\right)+1,$$

(18) $$m_{i}^{\left(c\right)}=mod\left(\left\lfloor 10^{6}k_{mask}^{\left(c\right)}(i)\right\rfloor ,4\right),$$

where $r_{i}^{\left(c\right)}$ selects one of the eight DNA rules in Table 1, and $m_{i}^{\left(c\right)}$ is a 2-bit mask. Each 8-bit pixel is divided into four 2-bit symbols $a_{i,j}^{\left(c\right)}$, where *j* = 0, 1, 2, 3, processed from the least significant pair to the most significant pair. A 2-bit feedback state *z* is maintained independently for each channel. Its initial value is *z* = $m_{1}^{\left(c\right)}$ ⊕ (*c* − 1), where ⊕ denotes bitwise XOR. For each symbol, the dynamic rule, mask, output symbol, and feedback state are updated by

(19) $$\left\{\begin{array}{c} r_{i,j}^{dyn,\left(c\right)}=mod\left(r_{i}^{\left(c\right)}-1+z,8\right)+1,\\ \mu_{i,j}^{\left(c\right)}=m_{i}^{\left(c\right)}⨁j⨁z,\\ y_{i,j}^{\left(c\right)}=T_{r_{i,j}^{dyn,\left(c\right)}}\left(a_{i,j}^{\left(c\right)}\right)⨁{\;\mu }_{i,j}^{\left(c\right)},\\ z\leftarrow y_{i,j}^{\left(c\right)}. \end{array}\right.$$

Within each channel, the feedback state *z* is initialized only once before the first pixel and is then carried continuously across all symbols and pixel boundaries. All operands in the XOR operations are restricted to two bits, and $T_{r}(\cdot )$ denotes the numeric mapping defined by the *r*-th DNA rule in Table 1. Internally, adenine (A), cytosine (C), guanine (G), and thymine (T) are represented by 0, 1, 2, and 3, respectively, so the DNA output can participate directly in 2-bit XOR operations. The four output symbols are recombined in least-significant-pair order as $D_{DNA}^{\left(c\right)}\left(i\right)=y_{i,0}^{\left(c\right)}+{4y}_{i,1}^{\left(c\right)}+16y_{i,2}^{\left(c\right)}+64y_{i,3}^{\left(c\right)}$. The standard mappings themselves are not claimed as new; the distinction lies in updating both the rule and mask for every 2-bit symbol according to the chaotic controls and the preceding transformed symbol. Consequently, a change in one input symbol modifies its own output and the state used by subsequent symbols. The ablation results in Section 4.10 provide empirical support for this mechanism.

**Figure 6:**
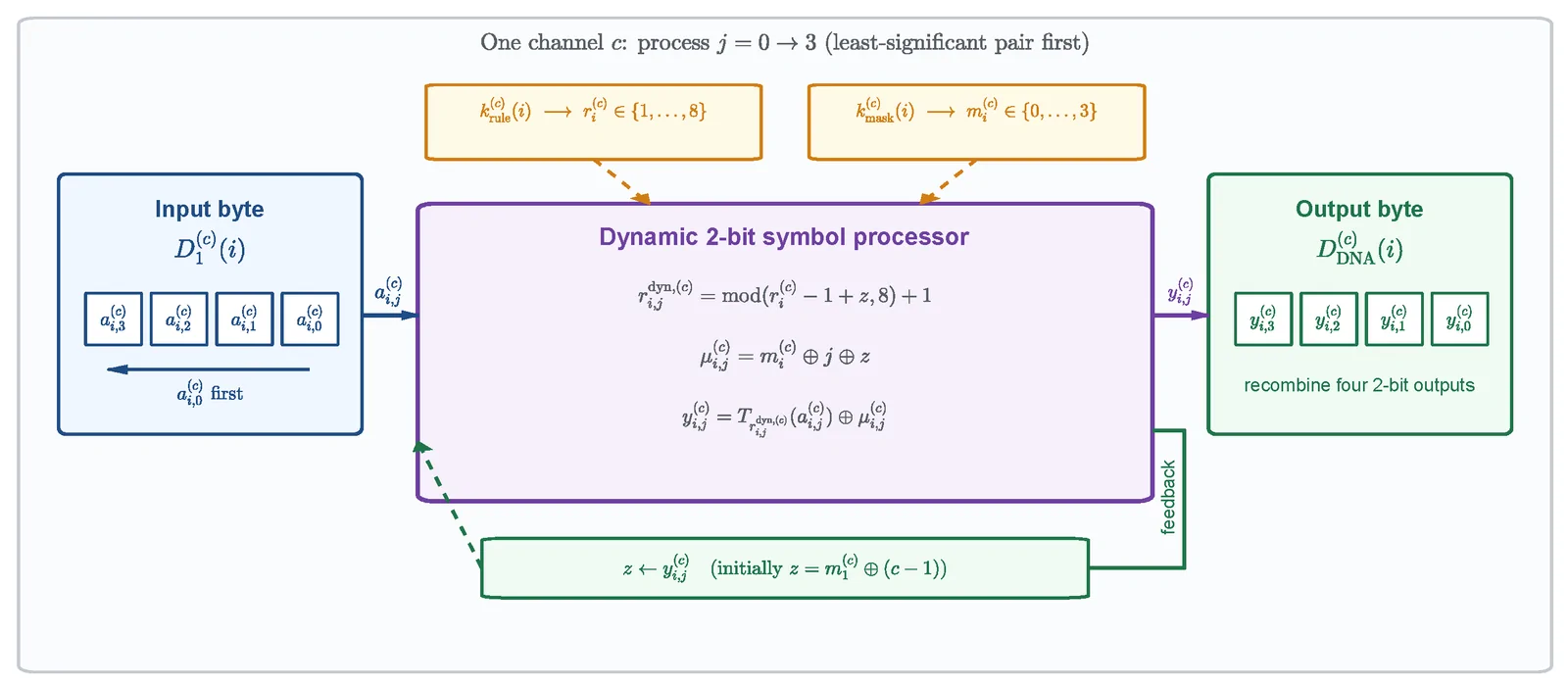
Dynamic DNA feedback mechanism for one color channel. The four 2-bit symbols of each byte are processed from the least-significant pair to the most-significant pair; each transformed symbol updates the feedback state for the next symbol, and the state is maintained across pixel boundaries. Dashed arrows denote control or state inputs to the symbol processor, whereas solid arrows indicate symbol flow and feedback-state updating.

The resulting symbol-level feedback process is illustrated in Figure 6.

Step 6: Secondary Permutation

A second key-dependent permutation is applied to *D*_DNA_ using *K_p_*_2_. For each color channel, the corresponding segment of *K_p_*_2_ is stably sorted in ascending order to obtain the integer permutation index vector *J_ch_*. The permuted sequence is obtained as *P*_2,*ch*_ = *D*_DNA,ch_(*J_ch_*). During decryption, the original ordering is recovered by the inverse assignment *D*_DNA,ch_(*J_ch_*) = *P*_2,*ch*_. This operation uses the same deterministic sorting convention as the initial permutation in Step 3.

Step 7: Secondary Bidirectional Chained Diffusion

Finally, a second bidirectional chained diffusion is applied to *P*_2_ using *K_d_*_2_. The second initialization vector is derived from the remaining three master-stream values as

(20) $$V_{2}=\left\lfloor 256[s_{6L+4,}s_{6L+5,}s_{6L+6}]\right\rfloor ,$$

where the floor operation is applied elementwise. The forward and backward recurrences are identical to those in Step 4, with *P*_2_, *K_d_*_2_, and *V*_2_ replacing *P*_1_, *K_d_*_1_, and *V*_1_, respectively. The output of this stage is the final ciphertext image *C*.

The forward and backward passes propagate changes in both processing directions, while the triangular R-to-G-to-B dependency provides cross-channel coupling with deterministic inversion. Placing the dynamic DNA operation between two permutation–diffusion stages and using six non-overlapping control streams avoids direct sequence reuse across stages. The key-dependent vectors *V*_1_ and *V*_2_ also eliminate fixed boundary states (Algorithm 1). The contribution of each stage is quantified by the ablation analysis in Section 4.10.
**Algorithm 1.** Encryption procedure**Input:** Plaintext image *P* ∈ {0, …, 255}^M×N×3^ and secret initial-state vector *K*_0_.
**Output:** Ciphertext image *C*.Set *n* = *MN* and *L* = 3*n*.Initialize the 6D system using *K*_0_, integrate it using fixed-step RK4 with *h* = 0.01, and discard the first 700 iterations.Generate 6*L* + 6 normalized chaotic values according to Equation (8).Divide the first 6*L* values into the non-overlapping streams *K_p_*_1_, *K_d_*_1_, *K*_rule_, *K*_mask_, *K_p_*_2_, and *K_d_*_2_.Derive *V*_1_ and *V*_2_ from the final six chaotic values according to Equations (14) and (20), respectively.Apply the initial permutation using *K_p_*_1_ to obtain *P*_1_.Apply bidirectional diffusion using *K_d_*_1_ and *V*_1_ to obtain *D*_1_.Apply the dynamic DNA transformation using *K*_rule_ and *K*_mask_ to obtain *D*_DNA_.Apply the secondary permutation using *K_p_*_2_ to obtain *P*_2_.Apply bidirectional diffusion using *K_d_*_2_ and *V*_2_ to obtain *C*.Return *C*.

### 3.2. Decryption Process

Decryption applies the inverse stages in reverse order using the same secret key *K*_0_, with modular subtraction and inverse DNA mappings replacing the corresponding encryption operations.

Step 1: Reconstruction of Chaotic Sub-key Sequences

Using *K*_0_, the receiver regenerates the master stream of length 6*L* + 6 and extracts *K_p_*_1_, *K_d_*_1_, *K*_rule_, *K*_mask_, *K_p_*_2_, and *K_d_*_2_ using the same non-overlapping segmentation defined in Step 2 of the encryption procedure. The vectors *V*_1_ and *V*_2_ are regenerated from the final six values of the stream.

Step 2: Inverse Secondary Diffusion and Permutation

First, the backward pass of the secondary diffusion is inverted using *K_d_*_2_ and *V*_2_, followed by inversion of its forward pass. In each inverse recurrence, modular addition is replaced by modular subtraction, while the ciphertext feedback values are read in the same order as in encryption. This recovers *P*_2_. The secondary permutation is then inverted by the assignment *D*_DNA,ch_(*J_ch_*) = *P*_2*,ch*_, yielding *D*_DNA_.

Step 3: Dynamic DNA Decoding

Each byte of the DNA-transformed intermediate image is divided into four 2-bit symbols in the same least-significant-pair-first order as during encryption. For each channel, the feedback state *z* is initialized once before the first pixel as $z=m_{1}^{\left(c\right)}⨁\left(c-1\right)$ and is then maintained continuously across pixel boundaries. For each received symbol $y_{i,j}^{\left(c\right)}$, the receiver regenerates $r_{i,j}^{dyn,(c)}$ and $\mu_{i,j}^{\left(c\right)}$ using the same chaotic control sequences and the current feedback state, and recovers the original symbol using

(21) $$a_{i,j}^{\left(c\right)}=T_{r_{i,j}^{dyn,\left(c\right)}}^{-1}(y_{i,j}^{\left(c\right)}⨁\mu_{i,j}^{\left(c\right)}).$$

After each symbol is recovered, the feedback state is updated as $z\leftarrow y_{i,j}^{\left(c\right)}$. The four recovered symbols are then recombined in least-significant-pair order to obtain the corresponding byte. After all three channels have been processed, the intermediate image *D*_1_ is recovered.

Step 4: Inverse Initial Diffusion and Permutation

The initial bidirectional diffusion is inverted using *K_d_*_1_ and *V*_1_, thereby recovering the initially permuted image *P*_1_. Finally, the initial permutation is inverted by the assignment *P_ch_*(*I_ch_*) = $P_{ch}^{\prime }$. The recovered channel vectors are reshaped to *M* × *N* arrays and combined to reconstruct the plaintext image *P* (Algorithm 2).
**Algorithm 2.** Decryption procedure**Input:** Ciphertext image *C* and secret initial-state vector *K*_0_.
**Output:** Recovered plaintext image *P*.Regenerate the master chaotic stream, six sub-key streams, permutation indices, *V*_1_, and *V*_2_ using *K*_0_.Invert the secondary bidirectional diffusion using *K_d_*_2_ and *V*_2_ to recover *P*_2_.Invert the secondary permutation using *K_p_*_2_ to recover *D*_DNA_.Apply dynamic DNA decoding using *K*_rule_ and *K*_mask_ to recover *D*_1_.Invert the initial bidirectional diffusion using *K_d_*_1_ and *V*_1_ to recover *P*_1_.Invert the initial permutation using *K_p_*_1_ to recover *P*.Return *P*.

## 4. Security Analysis

The following security analysis evaluates the proposed scheme in terms of pseudorandomness, statistical behavior, differential sensitivity, adjacent-pixel correlation, and effective key space.

### 4.1. Randomness Test

The digital generator was evaluated using all 15 categories of NIST SP 800-22 [41] with the official NIST STS 2.1.2 software (Table 2). One hundred initial states were independently sampled from the tested key intervals using the fixed seed 20260804. For each state, the system was integrated using fixed-step RK4 with *h* = 0.01 after discarding the first 700 iterations. Each state generated a sequence of 10^6^ bits, giving 10^8^ bits in total. At each iteration, the least significant eight bits of the quantized integer used in (8) were appended in most-significant-bit-first order.

**Figure 7:**
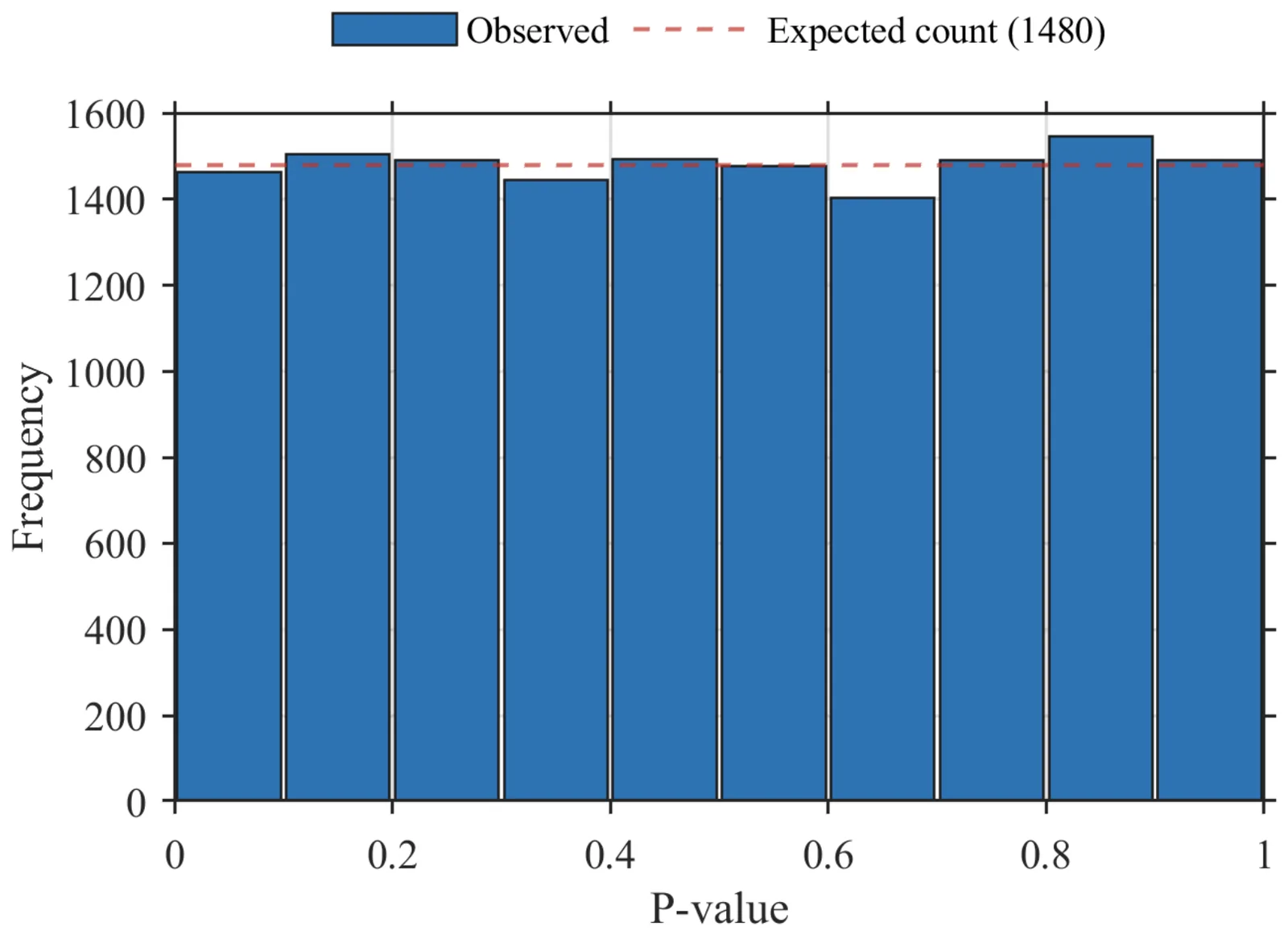
Distribution of 14,800 *p*-values from the NIST Non-overlapping Template test; the dashed line indicates the expected count of 1480 per bin.

All 15 test categories satisfied the uniformity and passing-proportion criteria. Among tests applicable to all 100 sequences, the minimum passing proportion was 0.960. The Random Excursions and Random Excursions Variant tests were applicable to 55 sequences and achieved minimum proportions of 54/55 and 53/55, respectively. The minimum uniformity *p*-value was 0.000600, exceeding the required threshold of 0.0001. Figure 7 further shows the 14,800 *p*-values from the 148 Non-overlapping Template tests, whose bin frequencies remain close to the expected count of 1480. These results support acceptable statistical randomness across the tested initial states.

### 4.2. Histogram Analysis

Histogram uniformity was quantitatively evaluated for the Baboon and Peppers images using histogram variance (HV) and the chi-square statistic. A smaller HV indicates a more balanced pixel-intensity distribution, while the chi-square statistic is defined by

(22) $$\chi^{2}=\sum_{i=0}^{255}\frac{{\left(O_{i}-E_{i}\right)}^{2}}{E_{i}},$$

where *O_i_* denotes the observed frequency of intensity value *i*, and *E_i_* = 1024 is the expected frequency for each intensity value in a 512 × 512 image channel.

According to Table 3, the plaintext histogram variances and chi-square values are large, whereas every ciphertext channel yields a chi-square value below the critical value of 293.2478 at the 0.05 significance level. The ciphertext *p*-values range from 0.1267 to 0.8898; therefore, the hypothesis of a uniform distribution is not rejected for any tested ciphertext channel. These quantitative results indicate that the encryption procedure substantially suppresses the observable pixel-intensity distribution of the tested plaintext images.

### 4.3. Information Entropy Analysis

Information entropy (IE) measures the uncertainty of pixel values. For an 8-bit image channel, its maximum value is 8 bits, and it is defined by

(23) $$H=-\sum_{i=0}^{255}p_{i}\log_{2}p_{i},$$

where *p_i_* is the probability of pixel value *i*. As shown in Table 4, the ciphertext-channel entropies range from 7.9992 to 7.9994 bits, close to the 8-bit maximum. Although entropy alone does not establish cryptographic security, it complements the histogram, correlation, differential, and key-sensitivity results. Table 5 further compares these entropy values with those reported in recent studies.

### 4.4. Differential Attack Resistance

NPCR and UACI quantify ciphertext changes resulting from a minute plaintext perturbation and are defined by

(24) $$NPCR=\frac{1}{3WH}\sum_{c=1}^{3}\sum_{i=1}^{H}\sum_{j=1}^{W}D_{c}(i,j)\times 100\%,$$

(25) $$UACI=\frac{1}{3WH}\sum_{c=1}^{3}\sum_{i=1}^{H}\sum_{j=1}^{W}\frac{\left|C_{1,c}\left(i,j\right)-C_{2,c}(i,j)\right|}{255}\times 100\%.$$

In the above equations, *H* and *W* denote the height and width of the image, respectively, and *c* ∈ {1,2,3} denotes the color channel. For an 8-bit random image, the theoretical expected values of NPCR and UACI are 99.6094% and 33.4635%, respectively.

Differential sensitivity was evaluated using six images, three secret initial-state keys, and ten independent trials for every image–key pair. In each trial, one randomly selected plaintext bit was flipped by sampling its pixel position, color channel, and bit position with the fixed seed 20260807. This produced 30 trials per image and 180 trials in total. As shown in Table 6, the overall NPCR and UACI were 99.6089 ± 0.0144% and 33.4554 ± 0.0456%, respectively, close to their theoretical expectations. The results demonstrate stable plaintext sensitivity across the tested images, keys, and perturbation positions. Table 7 further compares the NPCR and UACI results of the proposed scheme with those reported in recent studies.

### 4.5. Correlation Analysis

**Figure 8:**
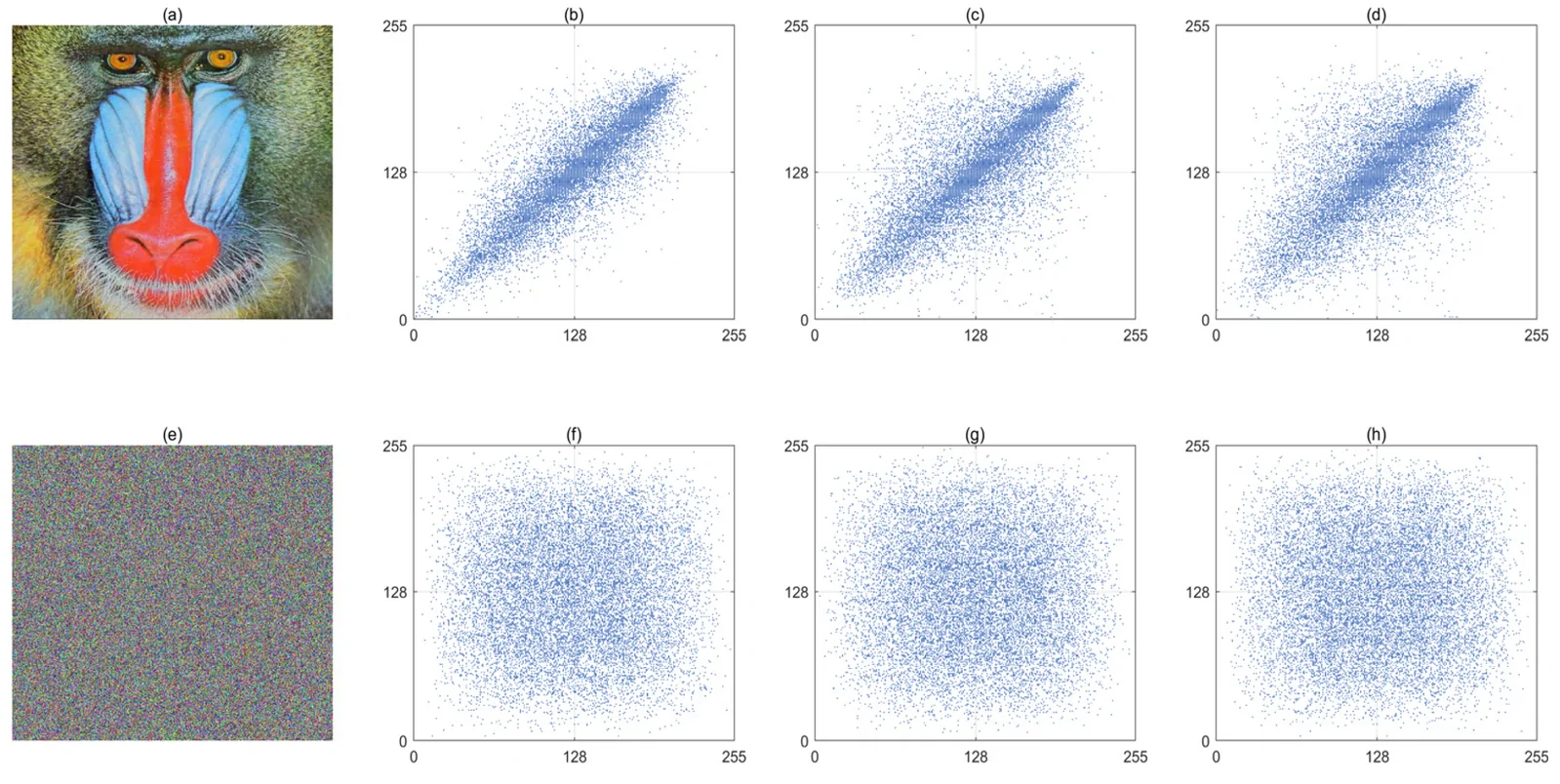
Adjacent-pixel correlations for the Baboon image: (**a**–**d**) plaintext and horizontal, vertical, and diagonal distributions; (**e**–**h**) ciphertext and corresponding distributions.

**Figure 9:**
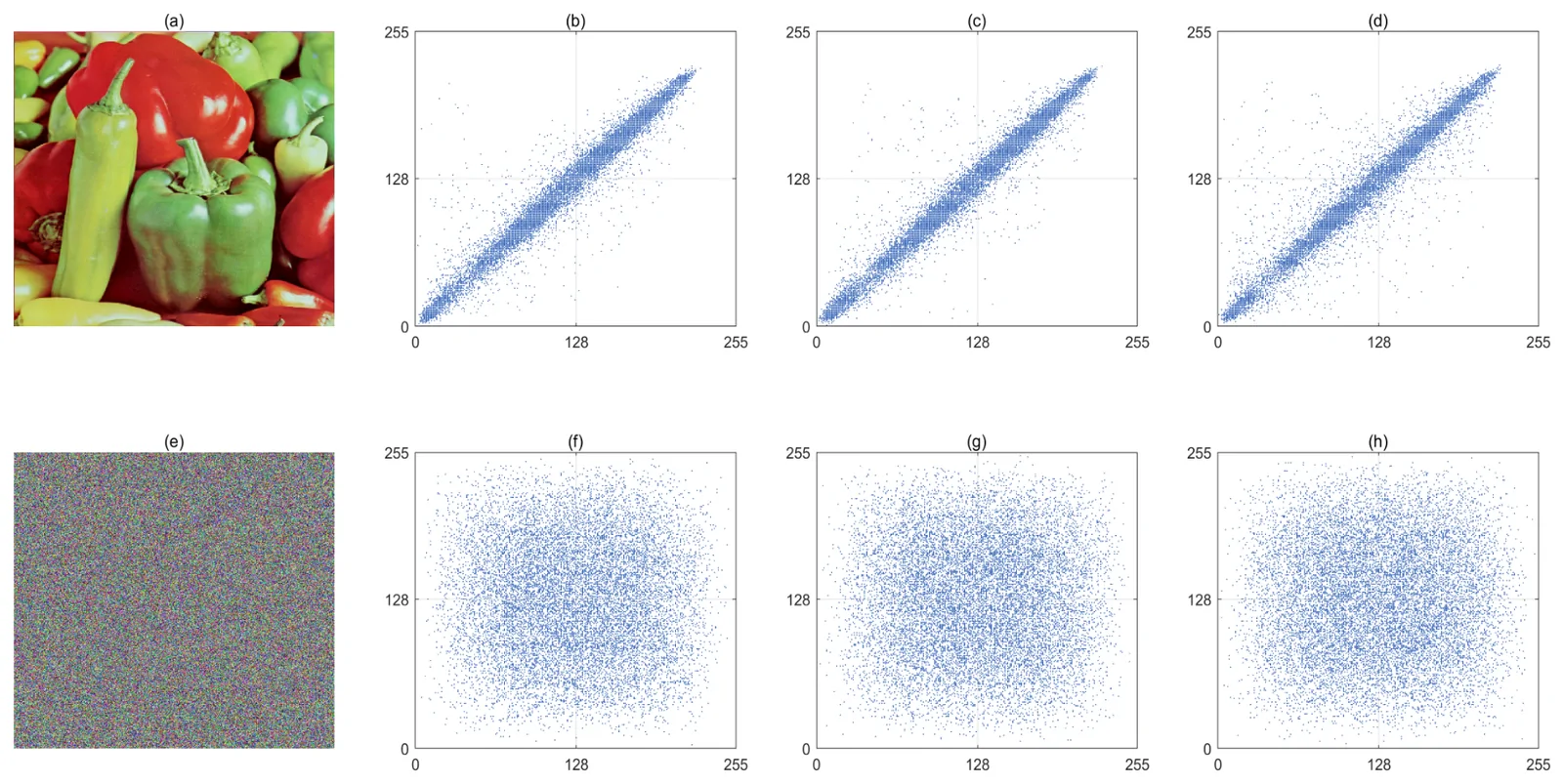
Adjacent-pixel correlations for the Peppers image: (**a**–**d**) plaintext and horizontal, vertical, and diagonal distributions; (**e**–**h**) ciphertext and corresponding distributions.

Adjacent-pixel correlation can reveal the spatial structure of a plaintext image and is quantified by

(26) $$r_{xy}=\frac{\sum_{i=1}^{N}\left(x_{i}-E\left(x\right))(y_{i}-E\left(y\right)\right)}{\sqrt{\sum_{i=1}^{N}{\left(x_{i}-E\left(x\right)\right)}^{2}\sum_{i=1}^{N}{\left(y_{i}-E\left(y\right)\right)}^{2}}},$$

where *x_i_* and *y_i_* are the intensities of the *i*-th adjacent-pixel pair, *N* is the number of pairs, and *E*(*x*) and *E*(*y*) are their mean intensities. Values of $\left|r_{xy}\right|$ close to zero indicate weak linear correlation. Table 8 uses all available adjacent-pixel pairs, while Figure 8 and Figure 9 visualize fixed random samples of 15,000 grayscale pairs per direction. The plaintext samples cluster near the diagonal, whereas the ciphertext samples are widely dispersed. The ciphertext coefficients range from −0.0051 to 0.0042, indicating substantial suppression of first-order spatial correlation. Table 9 further compares the ciphertext correlation coefficients of the proposed scheme with those reported in recent studies.

### 4.6. Key Space Analysis

The secret key consists only of the six initial conditions *K*_0_ = (*x*_0_, *y*_0_, *z*_0_, *u*_0_, *v*_0_, *w*_0_). The PSO-optimized coefficients and fixed implementation settings are public parameters, while *V*_1_ and *V*_2_ are deterministic functions of *K*_0_; none of these values introduces an independent key dimension. The tested intervals are *x*_0_, *y*_0_, *z*_0_ ∈ [0.5, 1.5], *u*_0_, *w*_0_ ∈ [−0.5, 0.5], and *v*_0_ ∈ [4.5, 5.5]. At a nominal resolution of 10^−14^, the key space is

(27) $$KeySpace={\left(10^{14}\right)}^{6}=10^{84}\approx 2^{279}.$$

This nominal space exceeds the conventional 2^128^ threshold but does not account for finite-precision, quantization, or equivalent-key effects. To examine digital distinguishability, 4096 bytes were generated for each of 20 sampled initial states using p*. All trajectories remained bounded and produced distinguishable streams. In the 120 cases obtained by perturbing each component by 10^−14^, the minimum raw-stream and byte-stream difference rates were 53.491% and 2.3438%, respectively. These finite-window results support 10^−14^ as a conservative nominal resolution without excluding equivalent keys beyond the tested sample.

### 4.7. Robustness of the Digital Sequence Generation Settings

The burn-in length, quantization gain, and state-aggregation method were evaluated using p*, ten initial states, and 50,000 output bytes per state and setting. Table 10 summarizes the byte entropy, absolute lag-one correlation $\left|\rho_{1}\right|$, and one-bit proportion.

Burn-in lengths from 0 to 1000 and gains from 10^6^ to 10^9^ produced nearly unchanged entropy and bit balance, indicating that the selected settings are not isolated values required for acceptable output. The aggregation methods also produced comparable results. Equal aggregation was retained because it treats all six states symmetrically without introducing an additional coefficient vector, although it is not claimed to be optimal. The final equal-weight configuration with gain 10^9^ also satisfies the IEEE 754 precision bound in Section 3.

### 4.8. Multi-Image and Multi-Key Evaluation

**Figure 10:**
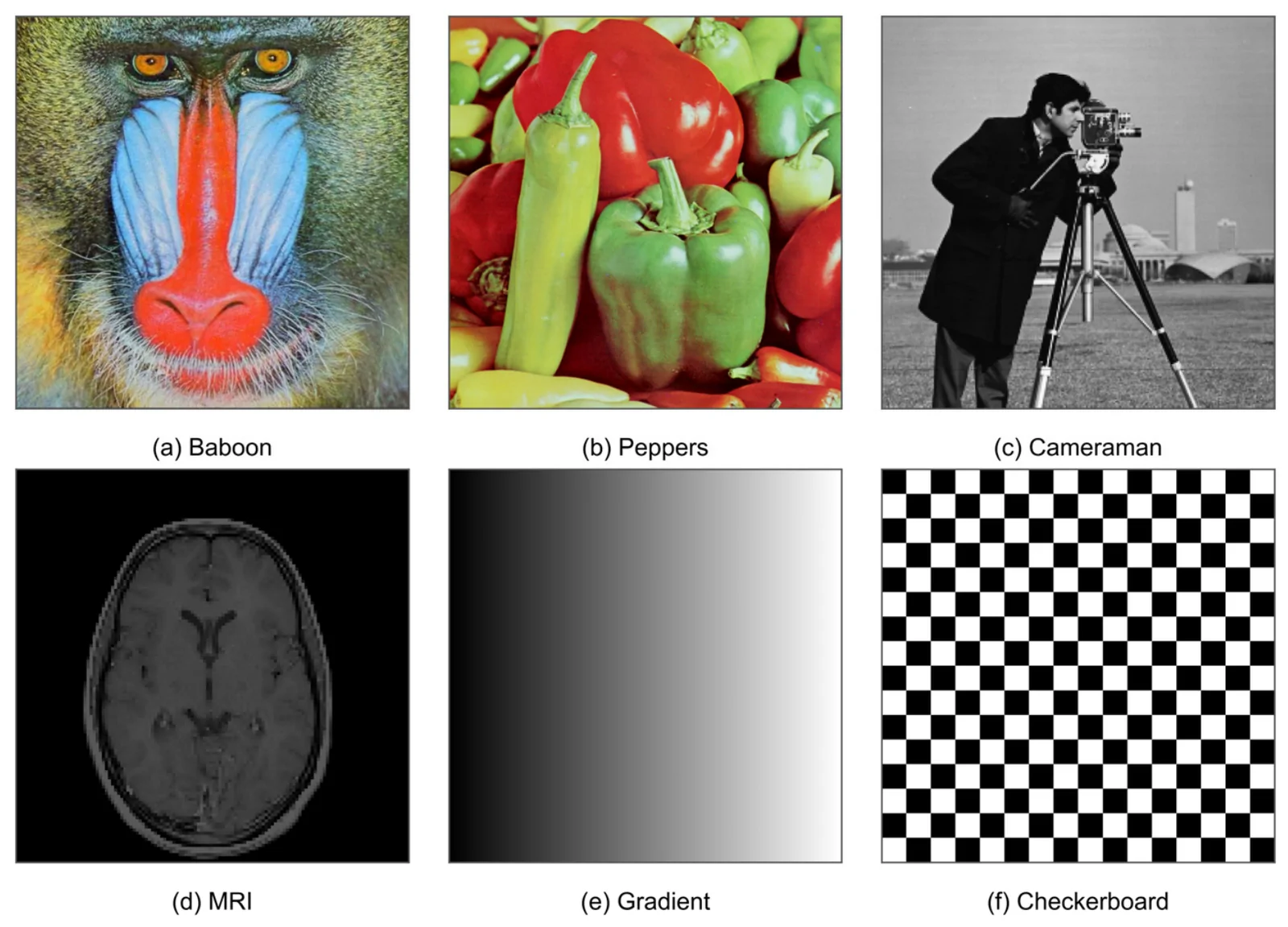
Test inputs used in the multi-image and multi-key evaluation: (**a**) Baboon; (**b**) Peppers; (**c**) Cameraman; (**d**) MRI; (**e**) Gradient; and (**f**) Checkerboard.

Six inputs with different structures, sizes, and modalities were evaluated: the 512 × 512 Baboon and Peppers color images, a 256 × 256 Cameraman image, a 128 × 128 MRI slice, a 384 × 384 gradient, and a 320 × 320 checkerboard. Grayscale inputs were represented by three identical channels to retain the same encryption pipeline. The test inputs are shown in Figure 10.

Three representative initial-state keys within the tested intervals specified in Section 4.6 were used, namely

(28) $$\left\{\begin{array}{c} K_{0}^{\left(1\right)}=\left(1,1,1,0,5,0\right),\\ K_{0}^{\left(2\right)}=\left(0.73,0.59,1.27,0.19,4.62,-0.38\right),\\ K_{0}^{\left(3\right)}=\left(0.88,1.12,0.63,0.31,5.22,0.17\right). \end{array}\right.$$

Each image was encrypted and decrypted using the three keys while all public parameters remained fixed. Exact recovery, PSNR, SSIM, ciphertext entropy, and the maximum absolute adjacent-pixel correlation were evaluated. Table 11 reports these results; the same keys were used in the differential experiments reported in Table 6 and in the ablation analysis described in Section 4.10.

All 18 image–key cases achieved exact pixel recovery, corresponding to an MSE of 0, a PSNR of +∞, and an SSIM of 1.0000. The overall entropy and maximum $\left|\rho \right|$ were 7.9969 ± 0.0039 and 0.00754 ± 0.00756, respectively.

### 4.9. Key Sensitivity Analysis

Key sensitivity was evaluated using the 512 × 512 Baboon image and the reference key *K*_0_ = (1, 1, 1, 0, 5, 0). Each initial-state component was independently perturbed by a nominal 10^−14^, with actual floating-point perturbations ranging from 9.77 × 10^−15^ to 1.00 × 10^−14^. Ciphertexts generated with the reference and perturbed keys were compared using NPCR and UACI, while wrong-key decryption was evaluated using PER, PSNR, and SSIM.

**Figure 11:**
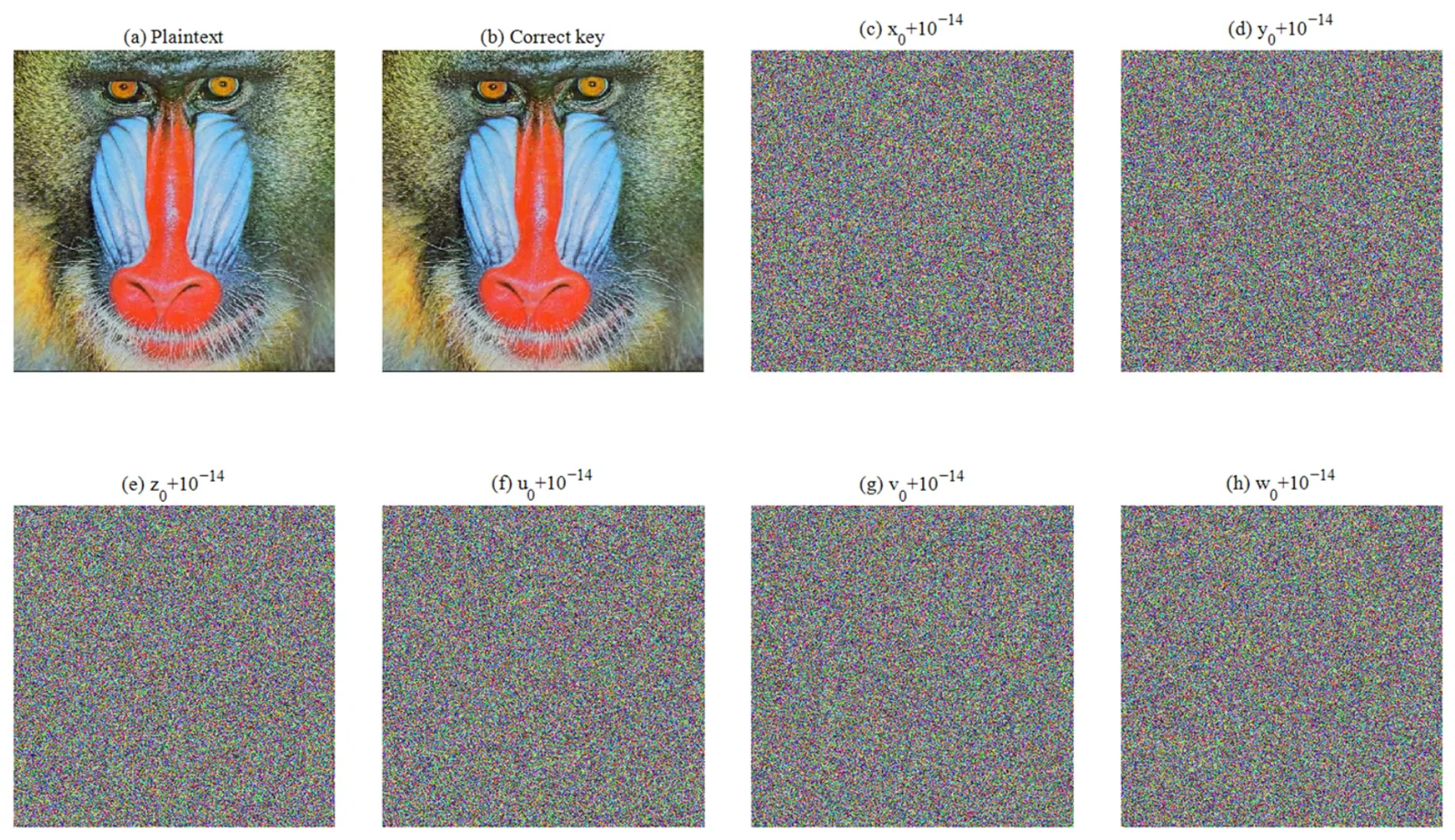
Key sensitivity results for the Baboon image: (**a**) plaintext image; (**b**) decrypted image obtained using the correct key; (**c**–**h**) decrypted images obtained by adding 10^−14^ separately to *x*_0_, *y*_0_, *z*_0_, *u*_0_, *v*_0_, and *w*_0_, respectively.

The correct key achieved exact recovery. As shown in Figure 11 and Table 12, every perturbed key produced a noise-like result, with PER values of 99.5964–99.6164%, PSNR values of 8.8171–8.8293 dB, and SSIM values of 0.009096–0.009771. The ciphertext NPCR and UACI values also remained close to their theoretical expectations, showing that all six initial-state components influence the encryption process.

### 4.10. Ablation Analysis

Eight configurations were evaluated using the Baboon and Peppers images and three initial-state keys: the complete scheme, five variants individually omitting one encryption stage, and two variants omitting both permutations or both diffusions. For each image, the same randomly selected one-bit perturbation was applied across all keys and configurations using the fixed seed 20260807. Table 13 reports the mean and standard deviation of entropy, maximum $\left|\rho \right|$, NPCR, and UACI over the six image–key cases.

Removing the dynamic DNA stage reduced NPCR to 75.0512%, while omitting the first or final diffusion reduced it to 87.4890% and 78.0984%, respectively. Without both diffusion stages, NPCR and UACI approached zero, confirming that chained diffusion is the principal mechanism for global avalanche propagation and that dynamic DNA strengthens this propagation. Removing permutation alone caused limited changes in these first-order metrics because the remaining stages continued to mask ciphertext statistics; nevertheless, permutation provides independent key-dependent spatial relocation.

### 4.11. Computational Complexity and Runtime Analysis

Let *n* = *M × N* denote the number of pixels in each color channel. The hyperchaotic sequence generator produces 18n + 6 samples, and therefore its computational cost is *O*(*n*). The two permutation stages require six sorting operations on length-n sequences, resulting in a complexity of *O*(*n* log *n*). The bidirectional chained diffusion and dynamic DNA operations perform a constant number of operations for each pixel or DNA symbol and consequently have a complexity of *O*(*n*). Therefore, the overall time complexity of both encryption and decryption is *O*(*n* log *n*), dominated by the sorting-based permutation stages, while the auxiliary memory complexity is *O*(*n*).

Runtime was measured in MATLAB R2024b on an AMD Ryzen 5 4600U computer with 15.4 GB of memory. Square top-left crops of the Baboon image from 128 × 128 to 512 × 512 were tested using $K_{0}^{\left(1\right)}=(1,1,1,0,5,0)$. Image loading was excluded, and three runs followed one unrecorded warm-up. All 15 runs achieved exact recovery. For the 512 × 512 image, the mean encryption and decryption times were 7.5562 s and 17.6760 s, respectively (Table 14).

For context, Zhou et al. [46] reported 3.0342 s and 3.3320 s, while Peng et al. [47] reported 260–283 s and 209–224 s for 512 × 512 RGB images. Hardware, software, and timing differences prevent a strict efficiency ranking. The current MATLAB prototype is not real-time; its main bottlenecks are numerical integration, sorting, symbol-wise DNA processing, and sequential feedback. Hardware acceleration would require fixed-point implementation, pipelined processing, and renewed security verification, as also suggested by the FPGA study in [15].

## 5. Conclusions

This study developed a color image encryption scheme using a PSO-optimized 6D Lorenz–Sprott hyperchaotic system. By optimizing the second-largest Lyapunov exponent, PSO identified a parameter set with two positive exponents that remained stable under reduced step sizes and ±1% individual parameter perturbations. The resulting sequences controlled two permutation stages, bidirectional cross-channel diffusion, and a symbol-level feedback-dependent DNA transformation. All 15 NIST SP 800-22 categories were satisfied across 100 independently initialized sequences, and exact reconstruction was achieved in all 18 image–key cases. Across 180 one-bit plaintext perturbations, the mean NPCR and UACI were 99.6089% and 33.4554%, respectively. Key-sensitivity and ablation results further confirmed the influence of all six secret initial states and the contributions of dynamic DNA and bidirectional diffusion.

The present work remains an experimental security evaluation rather than a formal cryptographic proof, and the current MATLAB implementation does not achieve real-time performance. Future work will therefore investigate hardware-oriented acceleration and dedicated analysis under known-plaintext and chosen-plaintext attack models. Additional sequence characterization will explore adapting the chaos game representation framework in [48] to the generated DNA-symbol sequences, while controlled comparisons with low-dimensional modulation-based generators such as the 2D Sine Logistic modulation map [49] will examine the trade-off between dynamical complexity and computational efficiency.

## Figures and Tables

**Table 1:** Eight standard DNA encoding and decoding rules.

Rule	1	2	3	4	5	6	7	8
00	A	A	T	T	C	C	G	G
01	C	G	C	G	A	T	A	T
10	G	C	G	C	T	A	T	A
11	T	T	A	A	G	G	C	C

**Table 2:** NIST test results.

No.	Statistical Test	*p*-Value	Proportion	Result
01	Frequency	0.983453	0.990	Pass
02	Block Frequency	0.001399	1.000	Pass
03	Cumulative Sums	0.202268	0.990	Pass
04	Runs	0.122325	0.970	Pass
05	Longest Run	0.971699	1.000	Pass
06	Rank	0.911413	1.000	Pass
07	FFT	0.574903	0.990	Pass
08	Non-overlapping Template	0.000600	0.960	Pass
09	Overlapping Template	0.719747	0.990	Pass
10	Universal	0.383827	0.980	Pass
11	Approximate Entropy	0.003201	1.000	Pass
12	Random Excursions	0.016717	0.982	Pass
13	Random Excursions Variant	0.007160	0.964	Pass
14	Serial	0.162606	0.990	Pass
15	Linear Complexity	0.935716	0.990	Pass

**Table 3:** Histogram variance and chi-square results.

Image	Channel	Type	HV	χ^2^	*p*-Value	Result
Baboon	R	Plaintext	293,507.44	73,376.86	<0.001	
		Ciphertext	1030.60	257.65	0.44	Pass
	G	Plaintext	584,121.71	146,030.43	<0.001	
		Ciphertext	992.41	248.10	0.61	Pass
	B	Plaintext	309,004.19	77,251.05	<0.001	
		Ciphertext	1123.88	280.97	0.13	Pass
Peppers	R	Plaintext	852,748.87	213,187.22	<0.001	
		Ciphertext	1059.71	264.93	0.32	Pass
	G	Plaintext	1,273,531.72	318,382.93	<0.001	
		Ciphertext	910.77	227.69	0.89	Pass
	B	Plaintext	1,965,712.71	491,428.18	<0.001	
		Ciphertext	988.53	247.13	0.63	Pass

**Table 4:** IE results for R, G, and B channels.

Image	R	G	B	Result
Baboon	7.9993	7.9993	7.9992	Pass
Peppers	7.9993	7.9994	7.9993	Pass

**Table 5:** Comparison of IE.

Reference	R	G	B
[42]	7.9971	7.9976	7.9975
[43]	7.9982	7.9988	7.9985
[44]	7.9993	7.9994	7.9992
[45]	7.9973	7.9974	7.9974
This paper	7.9993	7.9993	7.9992

**Table 6:** NPCR and UACI results.

Image	Size	Trials	NPCR (%)	UACI (%)
Baboon	512 × 512	30	99.6096 ± 0.0076	33.4654 ± 0.0238
Peppers	512 × 512	30	99.6085 ± 0.0074	33.4585 ± 0.0220
Cameraman	256 × 256	30	99.6112 ± 0.0138	33.4560 ± 0.0442
MRI	128 × 128	30	99.6049 ± 0.0256	33.4367 ± 0.0801
Gradient	384 × 384	30	99.6084 ± 0.0096	33.4488 ± 0.0327
Checkerboard	320 × 320	30	99.6109 ± 0.0141	33.4672 ± 0.0411
Overall	—	180	99.6089 ± 0.0144	33.4554 ± 0.0456

**Table 7:** Comparison of NPCR and UACI.

Reference	Channel	NPCR (%)	UACI (%)
[43]	R	99.5888	33.3231
	G	99.6088	33.3644
	B	99.6033	33.2469
[44]	R	99.6109	33.4533
	G	99.6162	33.4183
	B	99.5993	33.4823
[45]	R	99.5926	33.4897
	G	99.5900	33.4895
	B	99.5931	33.4911
This paper	R	99.6103	33.4613
	G	99.6088	33.4451
	B	99.6076	33.4600

**Table 8:** Correlation coefficients.

Image	Directions	Plain			Encrypted		
		R	G	B	R	G	B
Baboon	H	0.9285	0.8937	0.9366	0.0003	0.0005	−0.0018
	V	0.8648	0.8014	0.8839	−0.0051	−0.0009	−0.0021
	D	0.8423	0.7662	0.8621	−0.0027	−0.0004	−0.0039
Peppers	H	0.9635	0.9811	0.9665	−0.0012	0.0029	−0.0040
	V	0.9663	0.9818	0.9664	0.0003	−0.0009	−0.0004
	D	0.9564	0.9687	0.9478	−0.0050	0.0042	0.0005

**Table 9:** Comparison of correlation coefficients.

Reference	Channel	H	V	D
[42]	R	−0.0050	0.0047	−0.0021
	G	−0.0018	0.0027	0.0020
	B	0.0013	0.0024	−0.0061
[43]	R	−0.0037	−0.0027	0.0023
	G	0.0033	−0.0001	0.0084
	B	−0.0037	0.0116	0.0037
[44]	R	0.00014	0.0022	0.0024
	G	−0.0021	−0.0014	0.0021
	B	−0.0028	−0.0065	0.0015
[45]	R	0.0020	−0.017	−0.012
	G	0.0153	−0.0101	−0.0161
	B	0.0215	−0.0207	−0.0041
This paper	R	0.0003	−0.0051	−0.0027
	G	0.0005	−0.0009	−0.0004
	B	−0.0018	−0.0021	−0.0039

**Table 10:** Robustness of digital sequence-generation settings.

Parameter	Tested Settings	Entropy Range	Mean |*ρ*_1_| Range	One-Bit Proportion	Final Setting	Final-Setting Metrics
Burn-in length	0, 100, 300, 500, 700, 1000	7.996170–7.996195	0.016103–0.016693	0.49880–0.49886	700	7.996195; 0.016325; 0.498824
Quantization gain	10^6^, 10^7^, 10^8^, 10^9^	7.996195–7.996196	0.016325–0.016325	0.49882–0.49882	10^9^	7.996195; 0.016325; 0.498824
State aggregation	Equal; non-uniform; alternating; single-state; irrational	7.994674–7.996406	0.003170–0.184110	0.49527–0.49974	Equal	7.996195; 0.016325; 0.498824

**Table 11:** Multi-image and multi-key evaluation results.

Image/Modality	Size	Exact Recovery	PSNR	SSIM	Entropy	Maximum $\left|\rho \right|$
Baboon	512 × 512	3/3	+∞	1.0000	7.999274 ± 0.000046	0.003477 ± 0.001677
Peppers	512 × 512	3/3	+∞	1.0000	7.999295 ± 0.000024	0.003482 ± 0.001386
Cameraman	256 × 256	3/3	+∞	1.0000	7.997169 ± 0.000090	0.008817 ± 0.003475
MRI	128 × 128	3/3	+∞	1.0000	7.988528 ± 0.000459	0.021432 ± 0.009443
Gradient	384 × 384	3/3	+∞	1.0000	7.998816 ± 0.000079	0.004130 ± 0.000416
Checkerboard	320 × 320	3/3	+∞	1.0000	7.998222 ± 0.000081	0.003899 ± 0.000664

**Table 12:** Key-sensitivity results for individual initial-state perturbations.

Perturbed Component	Ciphertext NPCR (%)	Ciphertext UACI (%)	Wrong-Key PER (%)	PSNR (dB)	SSIM
*x*_0_	99.5973	33.4496	99.5964	8.8174	0.009448
*y*_0_	99.6025	33.4378	99.6142	8.8232	0.009520
*z*_0_	99.6124	33.4399	99.6119	8.8200	0.009187
*u*_0_	99.6164	33.4890	99.5996	8.8194	0.009771
*v*_0_	99.6106	33.4513	99.6164	8.8293	0.009096
*w*_0_	99.6012	33.4533	99.6033	8.8171	0.009719

**Table 13:** Ablation results (mean ± SD over six cases).

Configuration	Entropy	Maximum $\left|\rho \right|$	NPCR (%)	UACI (%)
Full scheme	7.99928 ± 0.00003	0.00348 ± 0.00138	99.6094 ± 0.0068	33.4441 ± 0.0319
Without initial permutation	7.99929 ± 0.00004	0.00366 ± 0.00149	99.6179 ± 0.0085	33.4699 ± 0.0204
Without first diffusion	7.99933 ± 0.00004	0.00359 ± 0.00105	87.4890 ± 28.8191	30.2237 ± 7.7588
Without dynamic DNA	7.99928 ± 0.00006	0.00289 ± 0.00048	75.0512 ± 0.0311	31.3866 ± 0.0161
Without second permutation	7.99931 ± 0.00003	0.00324 ± 0.00093	99.5441 ± 0.1654	33.3732 ± 0.1543
Without final diffusion	7.99930 ± 0.00001	0.00374 ± 0.00078	78.0984 ± 24.3979	27.9444 ± 8.8290
Without both permutations	7.99930 ± 0.00002	0.00361 ± 0.00069	99.6000 ± 0.2152	33.3734 ± 0.2217
Without both diffusions	7.98112 ± 0.01196	0.00368 ± 0.00094	0.0004 ± 0.0002	0.0002 ± 0.0001

**Table 14:** Runtime results and contextual comparison.

Scheme	Environment	Image Size	Encryption Time (s)	Decryption Time (s)
Proposed	MATLAB R2024b; AMD Ryzen 5 4600U; 15.4 GB	128 × 128	0.5968 ± 0.1890	1.2318 ± 0.2489
Proposed	MATLAB R2024b; AMD Ryzen 5 4600U; 15.4 GB	256 × 256	2.0032 ± 0.0766	4.2966 ± 0.1639
Proposed	MATLAB R2024b; AMD Ryzen 5 4600U; 15.4 GB	320 × 320	2.8722 ± 0.0890	6.4900 ± 0.0437
Proposed	MATLAB R2024b; AMD Ryzen 5 4600U; 15.4 GB	384 × 384	4.2058 ± 0.0309	9.4279 ± 0.1061
Proposed	MATLAB R2024b; AMD Ryzen 5 4600U; 15.4 GB	512 × 512	7.5562 ± 0.1034	17.6760 ± 1.4581
Zhou et al. [46]	MATLAB R2023a; AMD R9-7940HX, 2.40 GHz; 32 GB	512 × 512	3.0342	3.3320
Peng et al. [47]	MATLAB R2021b; Apple M1 Pro; 16 GB	512 × 512	260–283	209–224

## Data Availability

The original contributions presented in this study are included in the article. Further inquiries can be directed to the corresponding authors.
